# Correction of severe lower extremity deformity with digital hexapod external fixator based on CT data

**DOI:** 10.1186/s40001-022-00887-6

**Published:** 2022-11-17

**Authors:** Yufeng Lu, Jinfeng Li, Feng Qiao, Zhaochen Xu, Baogang Zhang, Bin Jia, Jinlong He, Liang Qi, Min Wang, Chen Fei, Xiaoming Cao

**Affiliations:** 1 Department of Integrated Traditional Chinese Medicine (TCM) and Western Medicine Orthopedics, Honghui Hospital, Xi’an Jiaotong University, Xi’an, 710054 Shaanxi People’s Republic of China; 2 Shaanxi University of Chinese Medicine, Xianyang, 712046 Shaanxi People’s Republic of China

**Keywords:** Multiplanar deformities, Hexapod external fixator, Electronic prescription, Gradual correction

## Abstract

**Purpose:**

Our goal was to examine the therapeutic effect of a self-designed digital six-axis external fixator technique for the correction of severe lower extremity deformities.

**Patients and methods:**

Between January 2017 and December 2020, our institution employed self-developed digital hexapod external fixator technology (QSF), based on CT data, to gradually correct 28 severe tibial deformities, and 15 femurs underwent osteotomy and internal fixation. The mean patient age was 32.6 ± 14.3 years, and the mean follow-up duration was 27.4 ± 16.1 months. We also recoded and analyzed the values of preoperative and final follow-up MAD, mFTA, MPTA, LLD, mLDFA, LEFS, KSS, and functional score.

**Results:**

The QSF adjustment duration was 21.4 ± 10.8 days, and the healing duration of the tibial osteotomy site was 17.6 ± 7.0 weeks. The preoperative MAD, mFTA, and MPTA were 54.1 ± 26.2 mm, 167.7 ± 15.7°, and 75.2 ± 12.0°, respectively. At the last follow-up, the MAD was 8.2 ± 9.9 mm, mFTA was 177.6 ± 3.4°, and MPTA was 87.6 ± 2.4°. Based on these data, we achieved significant improvement post operation. The preoperative LLD and mLDFA values were 13.8 ± 18 mm and 83.7 ± 10.8°, respectively, and the values were 7.6 ± 7.6 mm and 87.8 ± 2.6°, respectively, at the last follow-up. This indicated no significant difference in these values before and after the operation. Finally, the LEFS, KSS, and functional scores improved from preoperative 51.6 ± 11.2, 68.5 ± 11.7, and 67.8 ± 11.2 to postoperative 72.3 ± 6.1, 92.9 ± 3.4, and 94.2 ± 6.3, respectively.

**Conclusions:**

Based on our analyses, the QSF technique accurately corrected severe multiplanar tibial deformities in adults. When combined with femoral osteotomy, satisfactory lower extremity alignment was obtained while correcting for femoral deformity. This technology has the advantages of simple operation, reliable fixation, less trauma, and less complications.

## Introduction

Lower extremity deformities can result from trauma, as well as congenital, developmental, and degenerative diseases. The appropriate reconstruction of the normal lower extremity alignment is a key factor in obtaining satisfactory results after limb reconstruction. Severe lower extremity deformities are often combined with coronal, sagittal, and axial planes, as well as shortening deformities. As a result, orthopedic treatment becomes very challenging. Correction of lower extremity deformity using internal fixation alone generates a great impact on the bone blood supply, and it is necessary to ensure a successful one-time osteotomy, as postoperative adjustment cannot be performed. Alternately, the external fixation technique employs a “tension-stress” [1] effect and a "distraction osteogenesis" [2–4] mechanism to gradually correct the deformity, with less trauma and convenient postoperative adjustment [5, 6]. Therefore, using external fixation has great advantages over internal fixation in the correction of severe lower extremity deformities, and it has achieved great success in recent years. Lower extremity deformities can be corrected with monolateral or circular fixators. Monolateral external fixators are more comfortable and less bulky, but they have greater stiffness, and the eccentric stress is counterintuitive to fracture healing [7]. In addition, in many cases, they are not suitable for deformity correction [8]. The Ilizarov frame has a long learning curve, and requires the creation of additional hinges while correcting multiplanar deformities [8]. Moreover, its primary disadvantage is that it is particularly difficult to correct rotational deformity.

Relative to the Ilizarov frame, the computer-aided six-axis frames, such as Taylor spatial frame (TSF) can simultaneously correct coronal, sagittal, and axial deformities. Moreover, they are easy to use and have greater accuracy. Therefore, TSF has become the treatment of choice for multiplanar deformities in recent years [2, 3, 9–12]. However, the TSF-based correction of limb deformity must determine 13 total parameters: 3 frame, 4 mounting, and 6 deformity parameters. The measurement of each parameter produces errors that can affect the final orthopedic result. Moreover, the post-TSF adjustments depend on the precise anteroposterior and lateral radiographic measurements to produce the most accurate output [13, 14]. Hence, these X-rays must be taken in a plane orthogonal to the reference ring. Therefore, the X-ray measurements may also have errors due to shooting challenges. Moreover, the magnification of the X-ray will also affect the accuracy of the final correction.

In 2017, we developed a new six-axis spatial frame technology known as Qiao spatial frame (QSF), wherein the main structure was similar to TSF (Fig. 1). The difference between QSF and TSF was that the electronic prescription was calculated based on the CT, and not X-ray data. The aim of this study was to retrospectively analyze the clinical outcomes of severe deformities of the lower extremity treated with the QSF technique.

**Fig. 1:**
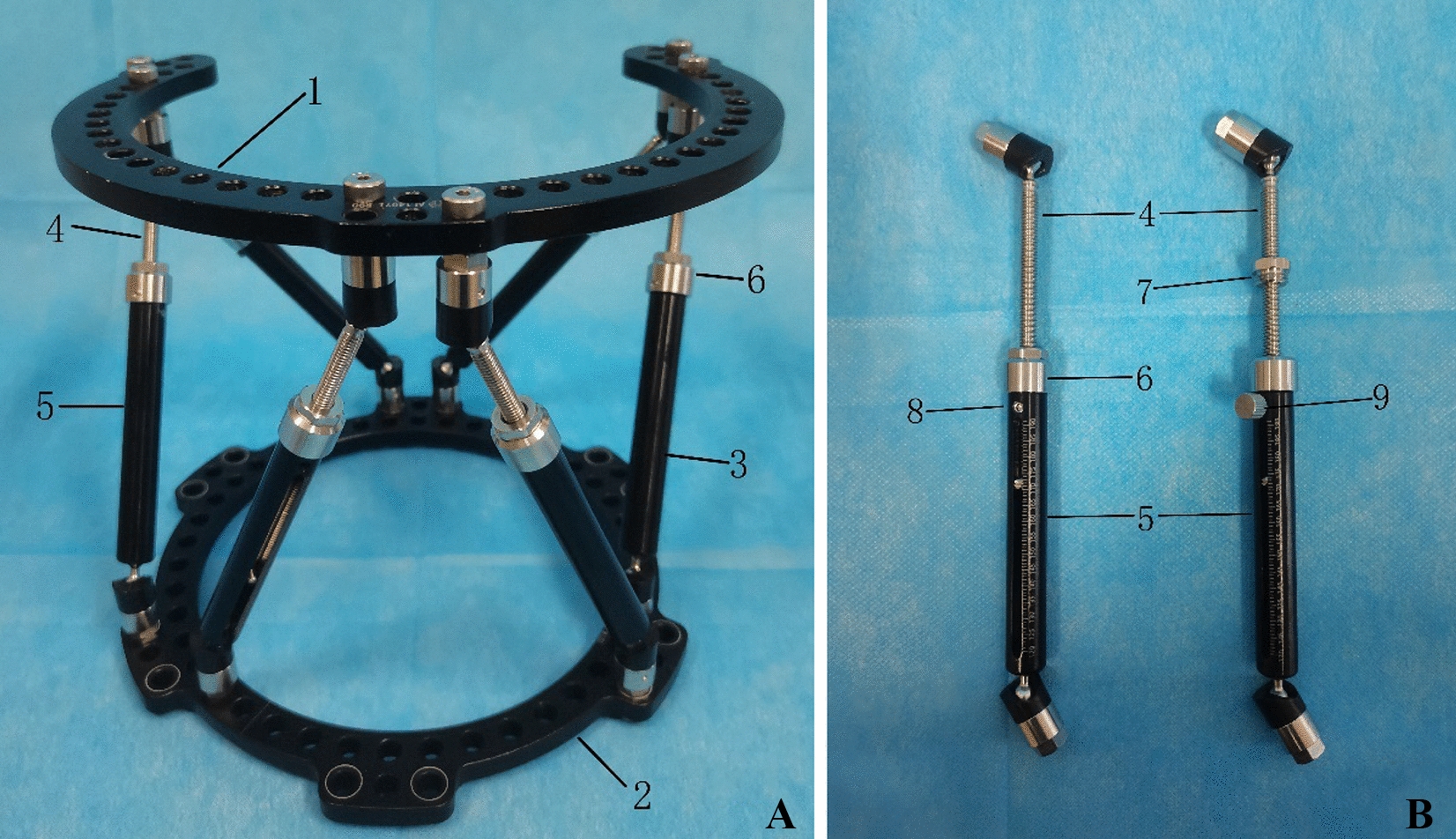
Qiao spatial frame (QSF) appearance photo (**A**) and connecting rod structure diagram (**B**). **1** 2/3 ring, **2** full ring, **3** strut, **4** inner rod, **5** outer rod, **6** adjusting nut, **7** small nut, **8** top screw, **9** manual top screw

## Materials and methods

Between January 2017 and December 2020, a total of 23 cases of lower limb deformities (31 legs) were treated with QSF external fixator at our institution. All patients presented with at least two deformities (angular and/or rotational, or shortening). Our exclusion criteria were as follows: (1) lower extremity deformities that occurred due to neurological or metabolic diseases. (2) presence of tumor.

One patient was eliminated from analysis due to the presence of multiple osteochondromatosis, and one additional patient due to spina bifida. Finally, 21 patients, with 28 lower extremities, were included for analysis. Among them, 10 were males, 11 were females, 16 suffered from left lower extremity deformities, and 12 suffered from right lower extremity deformities. The average patient age was 32.6 years (range 13–64 years). Out of the 21 patients, 13 had Blount's disease, 3 had rickets, and 5 had tibial fracture malunion. The mean follow-up duration was 27.4 months (Table 1).

**Table 1:** Summary of patient demographics

Characteristics	
Leg/patients	28/21
Ages (years)	32.6 ± 14.3
Male/female	10/11
Side (right/left)	12/16
Height (cm)	162.0 ± 11.8
Weight (kg)	64.0 ± 12.3
BMI (kg/m^2^)	24.2 ± 3.1
Follow-up (month)	27.4± 16.1
Diagnosis	
Blount disease	13
Rickets	3
Post-traumatic malunions	5

### Surgical technique

In the presence of femoral valgus deformity, and mLDFA greater than 90° or less than 85°, the femoral osteotomy was performed first. The apex of the angular deformity (CORA) was selected as the osteotomy site, and 4 osteotomies were carried out in the middle of the femoral shaft, and 11 in the distal femur. Prior to the operation, the Solidworks™ software was used to measure the correction angle, closed gap, distal femoral varus osteotomy selection of the distal femur anteromedial incision, and to expose the femoral distal medial cortex. The self-designed osteotomy guide sleeve [15] was placed close to the medial cortex of the distal femur, and five 2.5 mm Kirschner wires were drilled along the sleeve. The hinge position was confirmed via fluoroscopy, and the lateral hinge continuity was maintained during osteotomy. The bone wedge was removed, and the osteotomy gap was gradually closed until the medial cortex was completely fitted and fixed with the locking compression plate (TomoFix; Synthes, Solothurn, Switzerland). To perform valgus osteotomy in the middle of the femur, a lateral incision was made. The osteotomy guide sleeve was placed close to the lateral femoral cortex. Four Kirschner wires were inserted, and the osteotomy was performed along the direction of the Kirschner wires. Following removal of the bone wedge, the osteotomy gap was gradually closed, and fixed with locking compression plate (IRENE; Naton, Beijing, China).

Next, we performed fibula and tibial osteotomies. A longitudinal incision was made on the lateral side of the middle of the fibula, and a 1–2 cm long fibula was cut at the middle and lower 1/3 of the fibula. A partial transverse osteotomy was performed 1 cm below the tibial tubercle to facilitate the installation of an external fixator. A 2/3 ring was selected at the proximal end, and a full ring at the distal end. Based on the upper tibial deformity, the configuration of the six-axis external fixator was pre-adjusted. The adjusting nut on the connecting rod was loosened, and the top screw was tightened. At this time, the QSF configuration was basically stable. About 1–1.5 cm below the knee joint line, a 2.0 mm diameter Kirschner wire was placed from outside to inside, parallel to the tibial plateau, and the position of the proximal ring was adjusted and fixed, so that the opening direction of the 2/3 ring faced the rear to avoid affecting the knee joint flexion. Then, secure the proximal ring with the K-wire, loosen the top screw, at this time, the length of the connecting rod can be adjusted at will. Adjust the position of the distal ring to make it as perpendicular to the tibial axis as possible, then tighten the top screw on the connecting rod, and use 4 half pins to fix the distal ring. The proximal ring was fixed with two 2.0 mm diameter Kirschner wires and two half pins, or a 2.0 mm diameter Kirschner wire and three half pins. Fluoroscopy confirmed that the fixation was firm and the fixation pin was in a good position. Record the length of each connecting rod as the original length. Loosen the top screw on the QSF connecting rod, twist the two rings to make the osteotomy site completely fractured. Then reduced the osteotomy position and restored the length of each connecting rod to its original length.

Following the operation, we scanned the entire lower limb lengths using CT. The data were then entered into the supporting software to calculate the electronic prescription for six-axis external fixator adjustment. One week following the operation, the adjustment was initiated to slowly correct (speed: 0.7–1 mm/d) the deformities, based on the prescription. Following discharge, the patients adjusted the fixator themselves, according to the prescription. Upon the completion of deformity correction, the full-length AP weight-bearing X-ray of the lower extremity was taken. Based on the results, we determined the necessity for fine-tuning. Once the alignment adjustment was considered satisfactory, the strut was locked. X-rays were reviewed monthly, and the external fixator was removed after the osteotomy site had completely healed. During the correction process, the patient was instructed to perform knee and ankle range of motion exercises to prevent joint stiffness, and allow the affected limb to walk with tolerable weight-bearing activities.

### Radiographic measures

The lower limb alignment and leg length difference were recorded prior to the operation and after osteotomy site healing. The lower limb alignment was assessed via the mechanical axis deviation (MAD) and mechanical femorotibial axis (mFTA). The tibial alignment was evaluated by the medial proximal tibial angle (MPTA). Finally, the femoral alignment was assessed via the mechanical lateral distal femoral angle (mLDFA).

The lower limb length was recorded from the top of the femoral head to the center of the ipsilateral ankle joint on the anteroposterior standing whole-leg radiograph. The difference in the bilateral lower limb lengths was the leg length discrepancy (LLD). All measurements were conducted on the picture archiving and communication systems (PACS) (Synapse, Fujifilm Inc., Tokyo Japan). These measurements were carried out by 2 observers, who did not participate in the operation. After 3 weeks, the measurements were performed again by a single observer. The intraclass correlation coefficient (ICC) was applied to determine the reliability of all measurements. The ICC values were characterized as follows: poor agreement (< 0.40), fair to good agreement (0.40–0.75), and excellent agreement beyond chance (> 0.75).

### Clinical evaluation

The clinical outcome assessment was performed using the lower extremity functional scale (LEFS), KSS, and functional score before surgery and at the final follow-up.

### Statistical analyses

The SPSS version 26 (SPSS Inc., Chicago, IL, USA) was employed for all data analyses. The Kolmogorov–Smirnov test examined all dependent variables for normal distribution. The Paired t test was used to compare the MAD, mFTA, MPTA, LLD, mLDFA, LEFS, KSS, and functional scores prior to surgery and at the final follow-up. *P* < 0.05 was considered statistically significant.

## Results

Based on the Kolmogorov–Smirnov test, all data followed a normal distribution pattern. Moreover, the ICC and interclass correlation coefficients for the reproducibility of all parameters were > 80% (Table 2).

**Table 2:** Intraclass correlation coefficient analysis of four measured parameters before surgery and at the last follow-up

	Pre-MAD	Post-MAD	Pre-FTA	Post-FTA	Pre-MPTA	Post-MPTA	Pre-LLD	Post-LLD
Intraobserver	0.95	0.95	0.97	0.98	0.96	0.95	0.97	0.94
Interobserver	0.93	0.91	0.94	0.94	0.93	0.94	0.9	0.91

The average QSF-based adjustment time was 21.4 ± 10.8 d, and the time from the end of adjustment to osteotomy site healing was 17.6 ± 7.0 weeks. The preoperative MAD was 54.1 ± 26.2 mm, mFTA was 167.7 ± 15.7°, and MPTA was 75.2 ± 12.0°. At the last follow-up, MAD was 8.2 ± 9.9 mm, mFTA was 177.6 ± 3.4°, and MPTA was 87.6 ± 2.4° (Table 3). Relative to the preoperative values, the postoperative values showed significant improvement. The t values were 10.423, − 3.592, and − 5.866, respectively, all P < 0.01. The LLD went from preoperative 13.8 ± 18 mm to postoperative 7.6 ± 7.6 mm, showing no significant improvement after the operation (*t* = 1.859, *P* = 0.074). The mLDFA of the 15 femoral osteotomies went from preoperative 83.7 ± 10.8° to postoperative 87.8 ± 2.6°. Hence, relative to the preoperative values, there was no significant improvement in the mLDFA values (*t* = − 1.630, *P* = 0.125). The postoperative LEFS (72.3 ± 6.1) was significantly higher than the preoperative (51.6 ± 11.2) values (*t* = 12.285, *P* = 0.001). The KSS and functional score were significantly improved after the surgery (92.9 ± 3.4, 94.2 ± 6.3, respectively), compared to pre-surgery (68.5 ± 11.7, 67.8 ± 11.2, respectively) (*t* = 13.453, 14.554, respectively, both < 0.001).

**Table 3:** Radiological results before surgery and at the last follow-up

Case no	Side	Pre-MAD (mm)	Post-MAD (mm)	Pre-FTA (°)	Post-FTA (°)	Pre-MPTA (°)	Post-MPTA (°)	Pre-LLD (mm)	Post-LLD (mm)	Femoral osteotomy
1	Right	90.38	5.5	152	177	47	86	5.8	3.1	Yes
1	Left	82.14	4.9	156	180	57	86	5.8	3.1	Yes
2	Left	55.2	6	165	177	63	86	30.21	8	Yes
3	Right	75.4	33.6	157	170	66	82	6	15	No
3	Left	48.5	0	164	178	73	87	6	15	No
4	Right	74.2	5	150	178	68	94	4	5	Yes
4	Left	42	1.6	165	181	80	90	4	5	Yes
5	Right	42.6	0	165	179	66	88	2	16	Yes
5	Left	34.4	2.62	168	180	66	89	2	16	Yes
6	Left	39.1	5.7	169	180	73	86	8	6	Yes
7	Right	78.7	17.6	152	174	70	87	0.5	1.2	Yes
7	Left	71.5	5.5	152	179	72	87	0.5	1.2	Yes
8	Right	− 30.9	12.2	188	176	86	86	7	8	Yes
9	Right	59.6	16.3	164	177	70	87	27	8	Yes
10	Left	113.3	42.9	138	165	59	87	38	3	No
11	Left	67.1	7.6	159	177	70	88	9	0	No
12	Left	− 55.2	12.4	200	176	88	87	42.5	34	Yes
13	Left	36.2	0	171	180	81	90	5	0	No
14	Right	35.4	12.5	170	176	81	89	5	0	No
14	Left	33.1	0	170	179	81	91	5	0	No
15	Right	27.9	8.2	172	178	86	88	1	3	No
15	Left	22.7	11.3	173	178	86	88	1	3	No
16	Right	− 118.2	− 4	218	181	105	91	85	6.5	Yes
17	Left	47.2	0	167	180	79	89	8.5	12.5	No
18	Left	32	− 5	170	181	73	82	12	5	Yes
19	Left	27.6	2.9	171	180	81	88	13.5	6.8	No
20	Right	57.5	7.3	164	178	87	87	22.7	9.4	No
21	Right	− 17	0	186	180	92	87	30	20	No
Mean	–	54.1^△^	8.2^△^	167	177	75	87	13.8	7.6	–

Positive MAD value, mFTA<180°, indicating genu varus, negative MAD value, mFTA>180, indicating genu valgus. △was the result of statistics using the absolute number value

All patient wounds healed within 2 weeks after the operation, and the sutures were removed without nonunion, neurovascular injury, needle tract infection, osteomyelitis, and other complications. Only mild calf discomfort was reported in the later stage of the external frame adjustment. Typical cases of deformity correction are presented in Figs. 2 and 3.

**Fig. 2:**
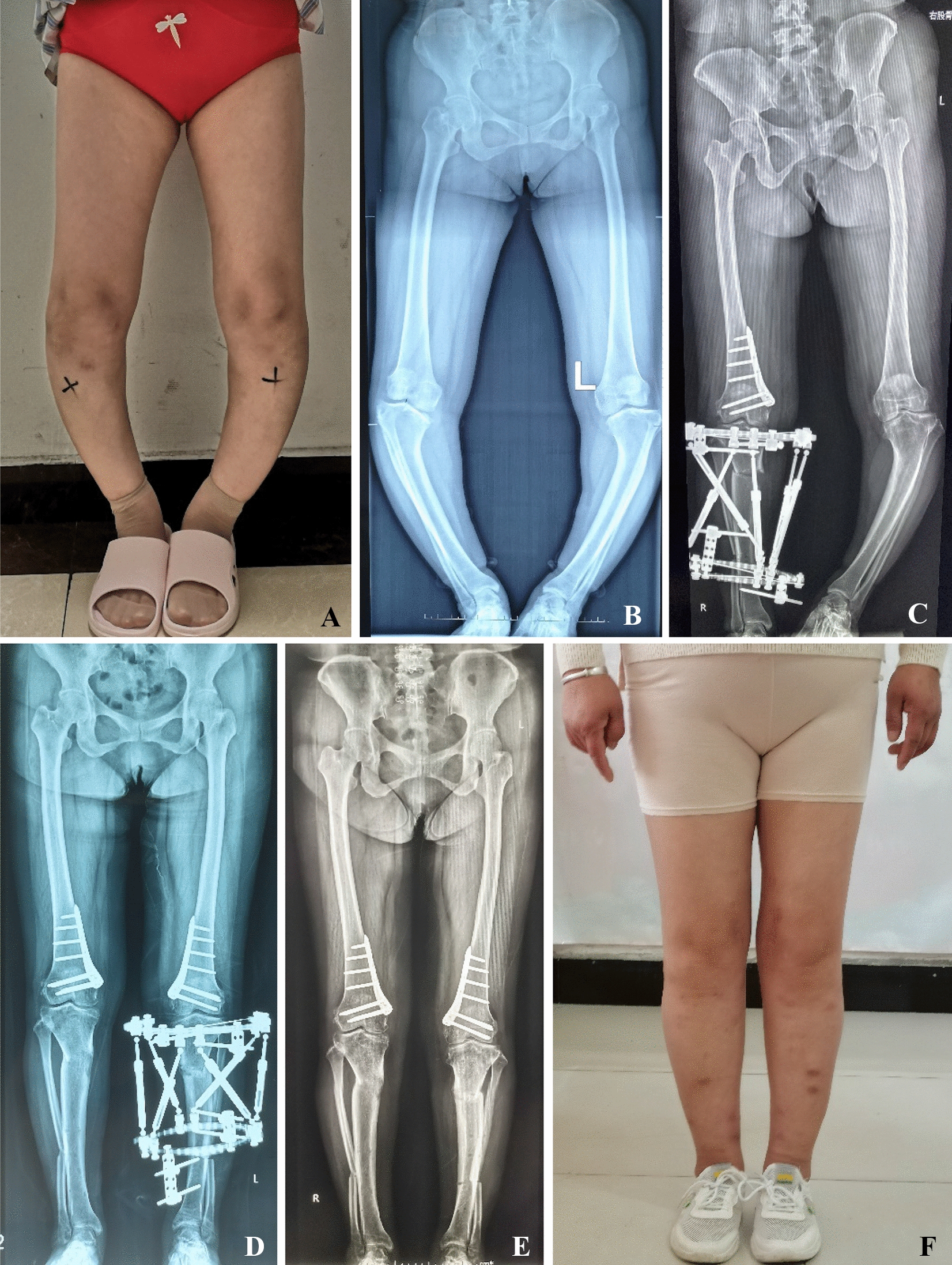
A 38-year-old female with juvenile Blount disease and untreated residual genu varus deformity. **A**, **B** Preoperative appearance and radiograph of lower limbs. **C** One-stage right tibial osteotomy QSF fixation and distal femoral osteotomy with internal fixation. The external frame was adjusted for 30 days, and the osteotomy site healed 15 weeks after the adjustment. At this point, the external fixator was removed. **D** Surgery was conducted on the left lower limb after 6 months. **E**, **F** Appearance and radiograph of the patient's lower extremities after the frames were removed

**Fig. 3:**
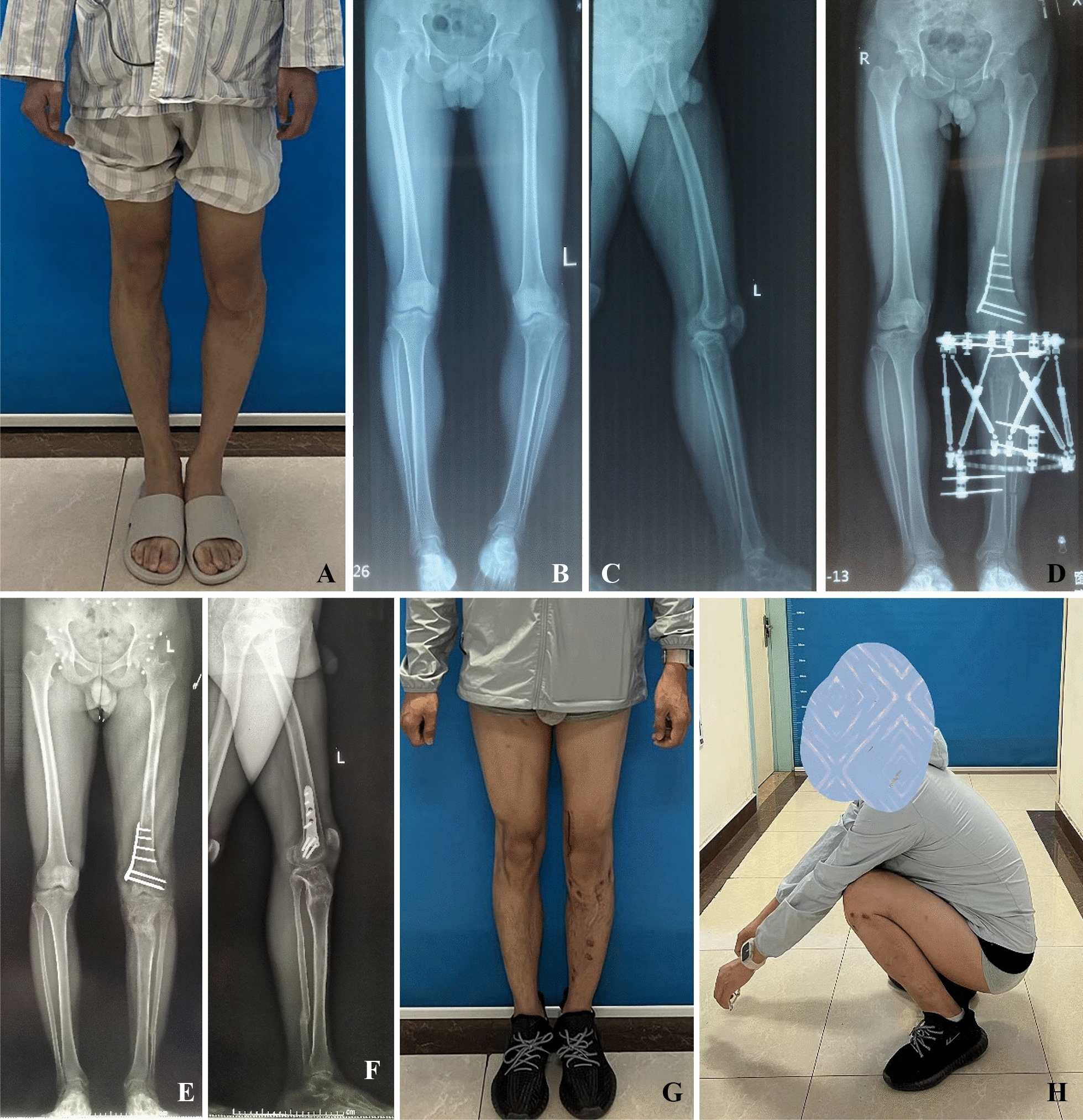
A 28-year-old male with juvenile Blount disease underwent osteotomy at the age of 14, and the deformity recurred 2 years later. **A**, **B** and **C** Preoperative appearance and radiograph of lower limbs. **D** One-stage left tibial osteotomy QSF fixation and distal femoral osteotomy with internal fixation. The external frame was adjusted for 45 days, and the osteotomy site healed 10 weeks after the adjustment. At this point, the external fixator was removed. **E**, **F**, **G** and **H** Appearance and radiograph of the patient's lower extremities after the frames were removed

## Discussion

Severe lower extremity deformities are often accompanied by coronal, sagittal, and axial deformities, and they are difficult to completely correct with a one-time internal fixation. In contrast, gradual correction can completely correct the three-dimensional deformity via tension–stress effects and distraction osteogenesis mechanisms. The single-arm external fixator is part of the eccentric fixation, and it cannot effectively resist the rotational stress and vertical deformation capacity. Relative to the acute correction of severe lower extremity deformities, gradual correction is, therefore, more attractive and effective [16].

The Ilizarov external fixator has been the standard versatile fixator for deformity correction for numerous years. Despite its many advantages, the correction of complex deformities using the Ilizarov frame requires a long learning curve, and the correction of rotational deformities still remains quite challenging [17]. In addition, when using Ilizarov, it is sometimes difficult to position the hinges in right locations. Although the Ilizarov frame could theoretically allow axial correction in every dimension, in practice, the treatment of multidimensional deformities may only be partially feasible, and, in most cases, all deformities can be treated incrementally. This not only increases the operation time, but also enhances the risk of future deformities [18]. Therefore, the correction of multiplanar and complex deformities with the Ilizarov frame is not recommended.

Relative to the Ilizarov technique, the computer-assisted hexapods are increasingly popular in deformity correction surgery, particularly, over the past few years. This is because of their ease of application and accuracy in correcting multiplanar deformities [17]. Ariyawatkul et al. compared the six-axis external frame and Ilizarov external frame in the treatment of tibial deformity, and concluded that the computer-assisted hexapods markedly reduced the Lengthening Index, compared to the conventional Ilizarov procedure. The hexapods device can be beneficial in achieving faster correction of complex deformity, if the patients or family members comprehend the protocols needed to manipulate the apparatus [19].

Reitenbach et al. evaluated 33 lower extremity deformities treated with TSF and 20 deformities corrected by the Ilizarov frame, and reported that the TSF axial deviation and pin infection were significantly less than those observed in Ilizarov-based fixation. The authors concluded that the TSF ring fixator produces fewer challenges, fewer secondary axial translations, and fewer pin infections  [11].

TSF is also effective in correcting femoral deformity. Hughes et al. [20] employed TSF to assist the correction of 49 femoral deformities, among which, 33 were multiplanar. Based on their analyses, the authors concluded that a six-axis external frame is a predictable and safe method for femoral deformity correction. In this group, the MAD, mFTA, and MPTA values were significantly improved after operation, compared to the preoperative values. The postoperative LLD was less than 2 cm, whereas the LEFS, KSS, and functional score were also significantly improved post operation.

Saw et al. [21] treated 22 patients with Blount's disease (32 varus knees) using Ilizarov and the TSF external frame. Based on their report, the pre- and postoperative MAD were 95 ± 51.4 mm and 9.0 ± 37.7 mm, respectively. The pre- and postoperative mFTA was 31 ± 15° varus and mFTA 2 ± 14° valgus, respectively. Sachs et al. [22] reported the use of the Taylor's frame in gradually correcting lower extremity deformities. In their study, the MAD value altered from preoperative 51.4 mm to postoperative 16.9 mm. Li et al. [23] reported a more severe deformity with a preoperative MAD value of 90 mm, and upon gradual correction, the postoperative MAD value reached 10 mm. In our study, the preoperative MAD value was 54.1 mm, and the postoperative MAD value was 8.2 mm, with an average correction of 47.9 mm.

The CT data-based QSF, developed by our institution, was composed of 6 mutually angled telescope struts and 2 connected plane rings, similar to the structure of the TSF. The ball-and-socket joint at both ends of the strut had no gap, and there was a top screw between the inner rod and the outer rod, so the overall QSF was very stable. In addition, its supporting software was based on the three-dimensional data of CT, so the precision was higher, and the precision of the prescription was 0.01 mm. Moreover, there were no special restrictions for the mounting position of the ring and the connection of the struts. Thus, the operation was simpler.

Since QSF was based on CT data, there was also no need to measure complicated parameters. The realignment result was not affected by the shooting angle of the X-ray film. Therefore, theoretically, the correction accuracy was higher than the X-ray-based TSF data. In addition, the advantages of QSF were that it had a short learning curve, was mastered without professional training, and was firmly fixed. In case of complex deformity correction, full weight bearing was achieved postoperatively.

The disadvantage of this study was the relatively small sample size and short follow-up time. Another limitation was that the radiograph measurement did not calculate the magnification rate. Based on the Sabharwal's study [24], the magnification rate of the standing full-length anteroposterior radiograph length value measurement was 1.05, which meant that our MAD and LLD data were likely 0.05 times larger than the actual value. Nevertheless, we speculate that the QSF technique can effectively treat the severe multiplanar deformity of the lower extremities in adults. However, it is necessary to increase the sample size and conduct long-term follow-up studies to validate our results.

## Conclusions

The QSF technology can accurately correct severe multiplanar deformity of the lower limb in adults. When combined with femoral osteotomy, QSF can be effectively used to correct lower extremity deformities, and to achieve satisfactory lower extremity alignment. This technology has the advantages of precision, simple operation, reliable fixation, short learning curve, less trauma and fewer complications. Therefore, it has a relatively high clinical application value.

## Data Availability

The data sets used and/or analyzed during the current study are available from the corresponding author on reasonable request.
